# Chitosan Modified Cationic Polyacrylamide Initiated by UV-H_2_O_2_ for Sludge Flocculation and New Insight on the Floc Characteristics Study

**DOI:** 10.3390/polym12112738

**Published:** 2020-11-18

**Authors:** Jie Chen, Xiaojun Xu, Rui Nie, Li Feng, Xuhao Li, Bingzhi Liu

**Affiliations:** 1 Faculty of Environmental Science and Engineering, Kunming University of Science and Technology, Kunming 650500, China; cj18669023333@sina.com (J.C.); xuxiaojun88@sina.com (X.X.); nr16kmlgdx8866@163.com (R.N.); 2 School of Civil and Transportation Engineering, Guangdong University of Technology, Guangzhou 510006, China; lixuhao_gdut2019@163.com (X.L.); 15922887515@163.com (B.L.)

**Keywords:** chitosan, UV-H_2_O_2_, grafting and copolymerization, sludge dewatering and conditioning, flocculation

## Abstract

In the present study, a novel graft modified flocculant CTS-g-PAMD was synthesized and applied to conduct sludge conditioning and dewatering. CTS-g-PAMD was copolymerized with AM, DMC and chitosan (CTS) under UV-H_2_O_2_ initiation. In addition, the effects of single factor experiments on the molecular weight (MW) CTS grafting efficiency (GE) of CTS-g-PAMD were determined and the optimal copolymerization conditions were achieved. The GE of CTS-g-PAMD reached 91.1% and the MW was 4.82 × 10^6^ Da. As revealed from the characterized results of Fourier-transform infrared spectra (FT-IR), ^1^H/ NMR, X-ray diffraction (XRD), scanning electron microscopic (SEM) and X-ray photoelectron spectroscopy (XPS), the successful synthesis of CTS-g-PAMD was confirmed, which is considered to be conducive to explaining sludge dewatering performance. Under the optimal conditions (pH = 7.0, flocculant dosage = 35 mg/L), the best flocculating performance (FCMC: 73.7%; SRF: 4.7 × 10^12^ m·kg^−1^, turbidity: 9.4 NTU) and large and dense sludge flocs (floc size d_50_ = 379.142 µm, floc fractal dimension D_f_ = 1.58) were formed. The DMC and CTS chain segments exhibiting cationic properties significantly improved the positive charge density and enhanced the electrical patching effect of CTS-g-PAMD. The long molecular chain of CTS-g-PAMD exhibited superior extensibility, which enhanced bridging effect on adsorption. Moreover, the sludge floc after undergoing CTS-g-PAMD conditioning exhibited robust shear resistance and regeneration ability. After the sludge floc was crushed and broken, a large and dense sludge floc was formed, helping significantly reduce the sludge specific resistance (SRF), turbidity and cake moisture content (FCMC) and enhance the sludge dewatering effect. The novel CTS-g-PAMD flocculant shows promising practical applications and high market value.

## 1. Introduction

As fueled by the leaping forward of the society, urban sewage and industrial wastewater are increasingly discharged and a rapidly growing number of sewage/wastewater treatments have been conducted. Biological method (activated sludge method) achieves extensive applications in sewage treatment; it acts as the technology primarily adopted to reduce organic pollutants in sewage. However, considerable activated sludge is generated in sewage treatment. High water content (90–98%) and large volume refer to one of the major water content characteristics exhibited by the mentioned activated sludge [1,2]. The sludge is dewatered to reduce its volume, which facilitates subsequent sludge treatment and disposal. Thus, sludge conditioning and dewatering are of critical significance [3,4]. Sludge acts as a colloidal system, in which small sludge particles distributed as stable suspension are difficult to separate from water phase [5]. Moreover, municipal sludge particles exhibit special floc structure and high hydrophilicity. For unconditioned sludge, the water contained in sludge is difficult to remove by conventional means (e.g., machinery) [6]. Sludge dewatering and conditioning by flocculation method are capable of effectively reducing the water content and the volume of sludge, thereby down-regulating the cost of sludge transportation. The mechanical dewatering performance exhibited by conditioned sludge is significantly enhanced to achieve sludge treatment and disposal.

Numerous inorganic and organic flocculants are being extensively used for their sufficient solid-water separation performance [7,8]. To be specific, the rapid development of natural modified polymer flocculants has aroused rising attention. Modified polymer flocculants are environmental-friendly, economical and efficient; their flocculation performance can be enhanced by graft modification. During sewage conditioning and dewatering, natural biopolymers should be synthesized and modified to be environmental-friendly and efficient sludge flocculants [9,10]. Chitosan (CTS) ranks as the second largest natural organic resource on the earth after cellulose. CTS, for its non-toxic, biodegradable and environmental-friendly properties, acts as an ideal raw material to synthesize flocculants for wastewater treatment and sludge dewatering [11,12]. Graft polymerization refers to a facile and simple method to prepare CTS modified flocculant [13]. Considerable active amino groups and hydroxyl groups exist in CTS skeleton, providing more active sites for grafting. Polymer materials exhibiting large MW can be grafted on CTS skeleton, thereby enhancing its flocculation performance [14,15]. It is generally known that the colloid in sludge has negative charge, as opposed to the cationic polyacrylamide (CPAM) band, which applies to charge neutralization. Moreover, its long polymer chain exhibiting the function of adsorption bridging can lead to relatively significant flocculating effect. Accordingly, CPAM grafting on CTS should be conducted to synthesize a novel CTS modified flocculant and alter its sludge flocculation and dewatering performance.

Acrylamide (AM) and cationic monomer methacryloyloxyethyl trimethyl ammonium chloride (DMC) refer to common monomers applied for synthesizing CPAM. Since they contain double constructs and exhibit high-soluble, low-cost, safe and environmental-friendly properties, they are easily initiated to polymerize, enabling them to achieve large MW and good flocculation performance [16,17]. It is thus suggested that AM and DMC act as polymerization monomers and are grafted onto CTS to prepare a novel flocculated CTS-P (AM-DMC), referred to as CTS-g-PAMD and used for sludge conditioning and dewatering. The main existing initiation polymerization methods consist of thermal initiation polymerization, ultraviolet UV-H_2_O_2_ microwave and ultrasonic initiation polymerization [8,18,19]. To be specific, UV-H_2_O_2_ initiation system is a more efficient and environmental-friendly method. UV-H_2_O_2_ is capable of producing hydroxyl radicals (•OH) by cracking H_2_O_2_ under ultraviolet light, thereby smoothly initiating monomer polymerization, increasing polymerization efficiency and producing no secondary pollution and so forth. It then has aroused growing attention. To be specific, the initiation method exhibits the advantages of s simple process, easy to control and master for operating conditions, short reaction time, less investment, environmental protection, as well as a low activation energy required for the reaction. Furthermore, UV-H_2_O_2_ polymerization can be rapidly initiated at ambient temperature and exhibits numerous advantages (e.g., low dosage of initiator, high efficiency, as well as health and safety), so it has been broadly advocated in recent years [20]. Accordingly, UV-H_2_O_2_ initiator can be applied for synthesizing new flocculated CTS-g-PAMD.

In the present study, AM and DMC were taken as monomers and UV-H_2_O_2_ initiation was adopted to graft CPAM based on CTS to prepare a high-efficiency flocculant CTS-g-PAMD for sludge dewatering. By single factor experiments, the polymerization law and rule of CTS-g-PAMD initiated by UV-H_2_O_2_ were preliminarily explored and the effect exerted by various single factor preparation conditions on the MW and CTS grafting efficiency was analyzed. By performing Fourier-transform infrared spectra (FT-IR), ^1^H/ NMR, X-ray diffraction (XRD), scanning electron microscopic (SEM) and X-ray photoelectron spectroscopy (XPS), the CTS-g-PAMD structure and its physical and chemical properties were characterized. The effects of initial pH, dosage and different types of flocculants on sludge conditioning and dewatering were investigated. The flocculation and dewatering properties of CTS-g-PAMD were assessed by filter cake moisture content (FCMC), specific resistance to filtration (SRF) and turbidity and a comparison was drawn between CTS-g-PAMD and commercial cationic polyacrylamide (CPAM). Furthermore, the correlation between the characteristics of sludge flocs and dewatering performance was studied by observing the particle size and fractal dimension of the generated sludge flocs, as well as by performing the flocs crushing and re-flocculation experiments. Given the analysis on zeta potential, the mechanism of sludge flocculation and dewatering was studied and summarized in depth.

## 2. Experimental Materials and Methods

### 2.1. Experimental Materials

AM was provided by Nanjing Kaida Chemical Co., Ltd. (Nanjing, China). Cationic monomer DMC (80 wt%) was donated by Suzhou Weidu Chemical Co., Ltd. (Suzhou, China). CTS (degree of deacetylation ≥ 95%, viscosity 100–200 mPa·s) originated from Zhengzhou Hongyitai Chemical Products Co., Ltd. (Zhengzhou, China). H_2_O_2_, as an initiator, was offered by Shanghai Huzheng Biotechnology Co., Ltd. (Shanghai, China). Ethanol (>99.7 wt%) and acetone (>99.5 wt%) were purchased from Chongqing Dongchuan Chemical Co., Ltd. (Chongqing, China). In addition, sodium hydroxide (>99 wt%) and hydrochloric acid (36 wt%) were provided by Dongming Chemical Co., Ltd. (Yantai, China), Muping District, Yantai City. Moreover, deionized water was adopted to prepare all aqueous solutions and standard solutions. N_2_ (purity 99.99%) employed in the experimental synthesis originated from Guangzhou Puyuan Gas Co., Ltd. (Guangzhou, China). Furthermore, AM and DMC were of industrial grade, the others were all of analytical grade. CTS-g-PAMD-2 (GE: 90.3%, MW: 5.0 × 10^6^ Da, CD: 29.6%), CTS-g-PAMD-1 (GE: 80.2%, MW: 4.0 × 10^6^ Da, CD: 28.7%) and CP(AM-DMC) shorted as PAMD (MW: 4.0 × 10^6^ Da, CD: 25.2%) were overall prepared by the authors in the laboratory.

### 2.2. Preparation of CTS-g-PAMD

**Figure 1:**
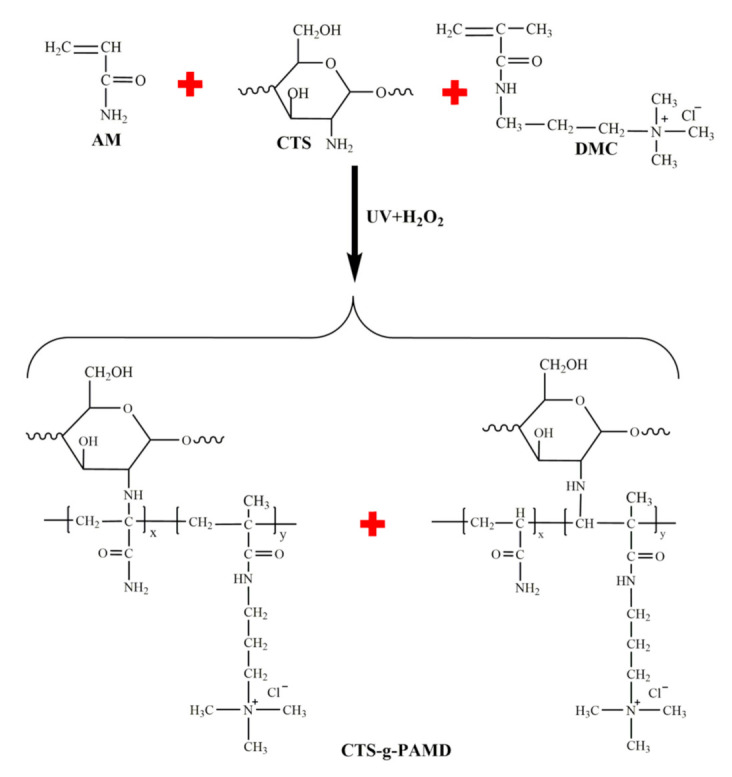
The reaction route of microwave assisted initiation of CTS-g-PAMD.

CTS with a predetermined amount was dissolved in 50 mL of 1.0% dilute hydrochloric acid solution to generate a CTS aqueous solution for standby. Subsequently, a certain amount of monomer AM, DMC and deionized water were added to the upper CTS aqueous solution while being constantly stirred till it was completely dissolved. The mass fraction of overall monomer (CTS + AM + DMC) was set to 20 wt%. The pH value of the reaction solution was regulated with 0.1 mol·L^−1^ HCl solution and 0.1 mol·L^−1^ NaOH solution. Next, N_2_ was charged into the reaction device for 20 min to overall expel oxygen from the reaction bottle. Initiator H_2_O_2_ with a certain amount was added immediately and the initiator was immediately placed into an ultraviolet light device (power: 160 W, λ = 365 nm) to initiate the copolymerization for 50 min. After the reaction was completed, the reaction bottle was taken out, stood and aged for 2 h. Afterwards, the product was rinsed in the reaction bottle with acetone and anhydrous ethanol for purification. After being washed several times, the product CTS-g-PAMD was generated by drying to a constant weight in a constant temperature oven at 70 °C. The synthesis method and steps polymer P(AM-DMC), abbreviated as PAD, were identical to those of CTS-g-PAMD. Moreover, the CTS-g-PAMD synthesis circuit is illustrated in Figure 1.

The cationic degree (DC) of the copolymer was determined by using colloid titration method. The polymer MW calculation method is presented in Supplementary Materials Text S1. The grafting efficiency (GE) of CTS was calculated by:

(1) $$Grafting\;\;efficiency\;\left(GE\right)\left(\%\right)=\frac{m-m_{CTS}}{m_{AM}+m_{DMC}}\;\times 100\%,$$

where *m* (g) denotes the total quality of the extracted and purified product; *m_CTS_* (g) represents the quality of the incoming CTS; *m_DMC_* (g) is the quality of the incoming DAC; *m_AM_* (g) is incoming AM mass.

### 2.3. Polymer Characterization

FTIR, XRD, ^1^H NMR, XPS and SEM of the polymerized products were investigated. The specific information is presented below. FTIR was performed on SeriesII 550 (Mettler-Toledo Instruments Co., Ltd. Shanghai, China) by employing KBr (potassium bromide) tabletting method under a wavelength range of 4000~400 cm^−1^. The ^1^H NMR spectra of the polymerized product were determined with Avance-500 NMR spectrometer manufactured by Bruker (Ettlingen, Germany). Heavy water (D_2_O) and tetramethylsilane (TMS) acted as the solvent and the internal standard, respectively. In addition, the XRD pattern of the polymer was recorded on an X-ray diffractometer (DMAX/2C, Shimadzu, Japan) equipped with Cu Kα radiation (λ = 1.54056 Å). Photoelectron spectroscopy spectra (XPS) were obtained with XPS spectrometer (ESCALAB250Xi, Thermo Fisher Scientific, Waltham, MA, USA) by taking Al-Kα X-ray as the excitation source. After gold was sprayed on the sample by Emitech sputtering ion coating instrument (Quorum Technologies, Ashford, UK), the morphological characteristics of the polymer sample to be tested were detected under MIRA 3 LMU scanning electron microscope (TESCAN, Brno, Czech Republic).

### 2.4. Sludge Flocculation Experiment

The sludge applied in the laboratory originated from a sewage plant in Guangzhou. Its property parameters are listed in Table 1. 500 mL of sewage was poured into a plurality of 1 L beakers and the pH value was regulated to the set value with HCl (0.1 mol/L) and NaOH (0.1 mol/L). The beaker was placed on a six-way mixer and a specific amount of flocculant sample solution was added to the beaker by complying with the experimental design to perform the sludge flocculation experiment. The flocculation experiment consisted of fast stirring for 60 s (400 r·min^−1^), slow stirring for 4 min (40 r·min^−1^) and standing for 15 min. Subsequently, the zeta potential of the sludge supernatant was determined with Zetasizer Nano ZS90 (Malvern Company, Malvern, UK). Moreover, the particle size and fractal dimension of sludge flocs produced by flocculation were measured. Such method is presented in Supplementary Materials Text S2 [21,22]. Next, the vacuum filtration dewatering experiment was performed on sludge to determine its FCMC and SRF. After that, a 2100P turbidity meter (HACH, Loveland, CO, USA) was used to determine the turbidity. The mentioned determination method is illustrated in Supplementary Materials Text S3 [11,23]. The particle size and fractal dimension of sludge flocs were also measured and calculated on a laser particle size distribution meter (BT-9300S, Beijing Judao Hesheng Technology Co., Ltd., Beijing, China). At the end of slow stirring (40 r/min) at the first phase, the broken and regeneration flocculation experiment was performed. The regeneration capacity, the particle size and the fractal dimension of sludge flocs after crushing and breakage were detected and recorded. During the experiment, the sludge floc particles were photographed under the computer-based transmissive polarizing microscope (XPV-600E) at certain intervals, while the images were processed by applying the image analysis software (Image-Pro Plus, version 6.0, Media Cybernetic). The sludge floc particles were stirred for 4 min (400 r/min) and slowly stirred for 15 min (40 r/min) and finally they stood for 20 min.

## 3. Results and Discussions

### 3.1. Single Factor Experimental Conditions

#### 3.1.1. Effect of Ultraviolet Power

**Figure 2:**
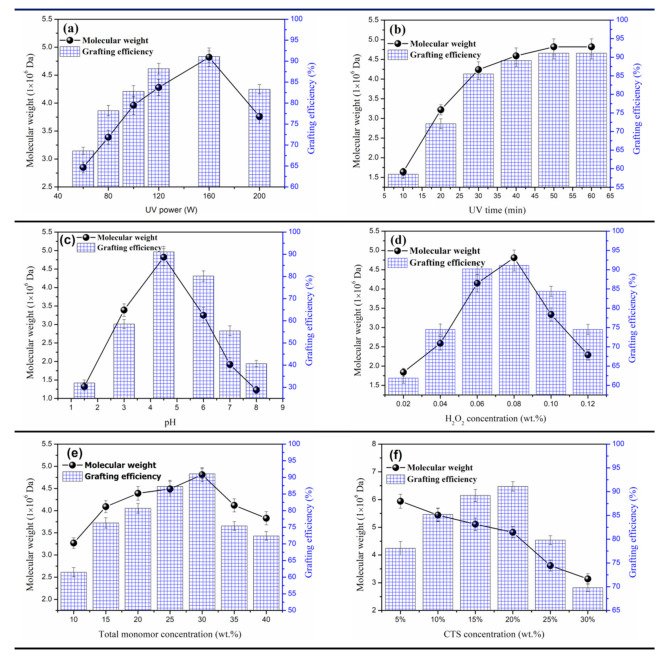
The effect of different single factors on the MW and chitosan (CTS) grafting efficiency of CTS-g-PAMD: (**a**) UV power, (**b**) UV time, (**c**) pH, (**d**) H_2_O_2_ concentration, (**e**) total monomer concentration and (**f**) CTS concentration.

Ultraviolet (UV) has been extensively applied in synthetic chemistry as an efficient and green initiation method. As impacted by UV light, H_2_O_2_ absorbs the energy of ultraviolet light, thereby leading to electron transition; such process can make H_2_O_2_ cracked and hydroxyl radicals (•OH) generated, which initiates monomer polymerization [24,25]. H_2_O_2_ acts as an initiator in the copolymerization. The polymerization effect is determined by the effect of UV radiation and UV power is considered one of the major factors of the radiation effect and H_2_O_2_ decomposition. Figure 2a indicates that the effect of UV light power on the polymerization reaction was investigated. Under the UV power of 160 W, the ME and conversion of the polymer were peaked, while higher or lower UV power restricts the polymerization reaction. This is because the energy generated by lower UV power fails to make H_2_O_2_ cracked rapidly and lead to the generation of sufficient initial free radicals; the monomer collision efficiency is low, resulting in lower polymerization MW and CTS grafting efficiency. As opposed to the above mentioned, when the power exceeded 160 W, too high UV power caused H_2_O_2_ to split rapidly to generate excessive free radicals, thereby causing severe collision of monomers, accelerating chain transfer and chain termination, as well as lowering MW and CTS grafting efficiency [26]. In addition, when monomers are being polymerized, considerable heat was released, so the liquid exhibited the local excessive temperature, which would lead to fracture and decomposition of the polymer main chain. Then, the MW of the polymer and the grafting efficiency of CTS would decline sharply. Based on the mentioned experimentally achieved results and analysis, the most suitable UV power selection is 160 W.

#### 3.1.2. Influence of Illumination Time

During the experiment, initiator H_2_O_2_ absorbed energy under UV radiation to generate primary free radicals, thereby initiating monomer copolymerization. Thus, the length of irradiation time would more significantly impact the degree of polymerization and the MW of the product. In the experimental process, other conditions were fixed constantly and the effects of UV irradiation time on the MW of the product and CTS grafting efficiency were studied. The results are illustrated in Figure 2b. As obviously shown in the figure, over the UV irradiation time, the MW of the product and CTS grafting efficiency rose to the maximum first and then decreased. This is because short-term UV irradiation cannot initiate the generation of sufficient active hydroxyl radicals to initiate monomer copolymerization. As a result, the reaction was insufficient, the CTS grafting efficiency was low, while the MW of the product was low. However, over the irradiation time, the number of active •OH in the system increased, the polymerization reaction turned more sufficient and both the MW of the product and the grafting efficiency of CTS were elevated [27,28]. When the irradiation time reached over a certain value (50 min in the experiment), the monomers capable of being involved in the reaction would be continuously consumed over the irradiation time and the reaction time and the excess UV irradiation energy would facilitate the disproportionation reaction and chain transfer reaction in the system; thus, the MW and CTS grafting efficiency of the product would be reduced. As a result, the most suitable UV irradiation time determined by this part of single factor experiment is 50 min.

#### 3.1.3. Influence of pH

In addition to the mentioned, the effect of the pH of the solution on the polymerization reaction was exacted and the results are presented in Figure 2c. With the pH regulated from 1.5 to 4.5, the MW and CTS grafting efficiency of CTS-g-PAMD increased and were peaked at pH = 4.5. Subsequently, the pH value rose continuously and the MW and CTS grafting efficiency decreased. This is because under the low pH of the system, the amide group on the acrylamide is prone to intermolecular or intramolecular imidization, thereby making the product cross-linked or branched chains generated and the MW of the product and CTS grafting efficiency were reduced [29]. However, under the relatively high pH, the quaternary ammonium salt group (-N^+^(CH_3_)_3_) on DMC monomer was hydrolyzed, which restricted the polymerization between monomers and reduced the MW of the product as well as the grafting efficiency of CTS. In addition, under relatively high pH values, the dissolution rate of CTS plummeted and CTS involved in the polymerization reaction would decrease significantly as well. Furthermore, the mentioned intolerant CTS would hinder the transmission of ultraviolet light, thereby reducing H_2_O_2_ decomposition efficiency and ultimately affecting the reduction of polymerization efficiency. Thus, the MW and CTS grafting efficiency of CTS-g-PAMD were reduced. In this experiment, pH = 4.5 was taken as the appropriate reaction condition.

#### 3.1.4. Effect of H_2_O_2_ Initiator

Radicals generated by initiator H_2_O_2_ could initiate AM, DMC and CTS monomers to generate chain radicals and induce polymerization to generate CTS-g-PAMD. H_2_O_2_ could critically impact the UV-H_2_O_2_ initiation system and significantly impact polymerization. In this study, the effect of H_2_O_2_ concentration on the MW of the polymerized product and CTS grafting efficiency was investigated. The experimentally achieved results are illustrated in Figure 2d. On the whole, the MW and CTS grafting efficiency of CTS-g-PAMD decreased with the increase in the initiator concentration. Under the H_2_O_2_ concentration of 0.08 wt%, the MW and CTS grafting efficiency of CTS-g-PAMD were the maximum. This is because H_2_O_2_ initiator acts as the active center for free radical generation and controls the polymerization process. Under the excessively low concentration of H_2_O_2_, the number of •OH generated by UV initiation was small and the collision probability between •OH and monomers was low, adversely affecting the growth of polymerization chain and hindering the chain initiation, which extended the polymerization time and reduced the MW of the product [30]. With the increase in H_2_O_2_ concentration, the amount of active •OH generated by ultraviolet initiation was up-regulated, the initiation rate of such radical increased and the polymerization reaction proceeded rapidly and completely; thus, the MW and CTS grafting efficiency of the product were elevated as well. However, under the concentration of H_2_O_2_ reaching over the optimal concentration and even higher, the number of •OH yield was too large, significantly elevating the number of active centers in the polymerization reaction. Under random collisions between the mentioned active hydroxyl radicals, chain transfer and chain termination of the polymerization side reaction were more likely to take place, so the MW and CTS grafting efficiency of the product began to decrease again [31]. Given the mentioned analysis, this part of the single factor experiment determined the optimal H_2_O_2_ concentration dosage concentration as 0.08 wt%.

#### 3.1.5. Influence of Mass Fraction of Overall Monomers

The overall monomer content in the polymerization system largely determines the number of free monomer radicals, affecting the polymerization rate, polymer MW and CTS grafting efficiency. Accordingly, the effect of the change of overall monomer mass fraction on polymer MW and CTS grafting efficiency should be investigated. The test results are presented in Figure 2e. With the increase in overall monomer mass fraction, the MW of polymer, CTS-g-PAMD and CTS grafting ratio increased first and reached the peaks of 4.82 × 10^6^ Da and 91.1%, respectively, when the monomer mass fraction was 25% and then they tended to decrease. This is because the mass fraction of the overall monomer at the beginning of the reaction determines the number of free radicals generated; when the number of free radicals is small, “cage effect” will occur, resulting in the early termination of the polymerization reaction [27]. Moreover, the monomer collision was positively correlated with the overall monomer content and the lower overall monomer content hindered the occurrence of chain growth reaction, resulting in lower intrinsic viscosity and conversion rate. With the increase in the mass fraction of overall monomers, the “cage effect” was weakened, free radicals were continuously generated, AM, DMC and CTS were more likely to collide with each other and copolymers were continuously synthesized via chain growth reaction, so the CTS grafting efficiency and MW of CTS-g-PAMD were improved. However, the overall monomer was too high and the dissolution effect of monomer was reduced, which limited the efficient reaction. In addition, an extremely high total list will produce excessive chain free radicals and the polymerization reaction would be overly intense, thereby ending the chain growth reaction in advance and leading to the chain termination reaction. In addition, excessive chain radical violent reaction would produce considerable polymerization heat that is difficult to dissipate and the rise of polymerization temperature would reduce the polymerization degree of the product, eventually causing chain termination and chain transfer reactions. Thus, when the mass fraction of the overall monomer increased to 30 wt%, the MW of the product and the grafting efficiency of CTS began to decrease. Accordingly, the optimal mass fraction of overall monomers was set to 30 wt%.

#### 3.1.6. Influence of Mass Fraction of CTS Monomer

Figure 2f indicates that the MW and CTS grafting efficiency of CTS-g-PAMD gradually decreased with the increase in CTS mass fraction. When m_CTS_:(m_Total Monomors_) = 20%, the MW decreased slowly. Then, the grafting efficiency was peaked. The activity of AM and DMC monomers was more active than that of CTS and the steric hindrance of monomers participating in the reaction was less than that of CTS. Under the low content of CTS in the solution, the reaction rate of AM and DMC with higher contents in the reaction system was elevated, resulting in higher MW of the product. Under the gradually increasing mass ratio of CTS, the MW of CTS-g-PAMD decreased rapidly due to the high energy barrier required for CTS -NH_2_ grafting AM and DMC at C2 site and the olefin bond between AM and DMC molecules turned out to be easy to break and polymerize. In addition, CTS exhibiting a large MW was easy to produce spatial staggered grafting reaction during polymerization, resulting in steric hindrance effect and affecting the approach of monomer to the free radical end of molecular chain. Accordingly, the growth of molecular chain was hindered and the MW of the product was reduced. Moreover, the appropriate amount of CTS content helped provide sufficient graft polymerization active sites -C2–NH_2_ and it facilitated AM and DMC monomer grafting. Thus, the optimum m_CTS_:(m_Total Monomors_) = 20% was selected here. The MW of polymer and CTS adhesion efficiency are both appropriate. CTS-g-PAMD exhibited high adsorption bridging and electric neutralization effects and the prominent flocculating effect was achieved.

After a comparison of the results from previous literature [11,32,33], it was found that the UV-H_2_O_2_ initiation can be employed to prepare the graft modified flocculant CTS-g-PAMD efficiently. The initiation and polymerization effect of UV-H_2_O_2_ is desirable and advisable.

### 3.2. Characterization

#### 3.2.1. FTIR Analysis

**Figure 3:**
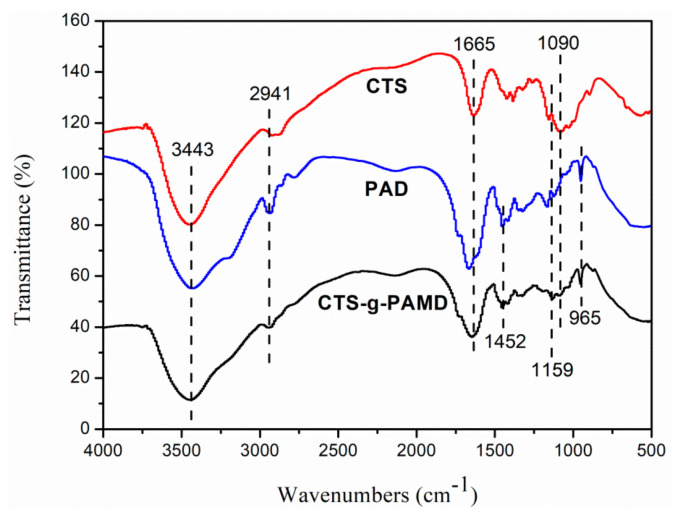
The Fourier-transform infrared spectra (FT-IR) of the polymers (AM, DMC and CTS).

The FTIR of polymers CTS-g-PAMD, PAD and CTS were investigated to gain insights into their functional group structures. The results are presented in Figure 3. For CTS, the absorption peaks at 1665 cm^−1^ were assigned to the bending vibration of C–H from -CH_3_ on CTS. The absorption peaks at 1159 cm^−1^ were derived from the bending vibration of N–H on CTS. The bending vibration of C–O–C asymmetric deformation vibration and the stretching vibration of C–OH were all observed at 1090 cm^−1^ [14,26]. Moreover, the deformation vibration peaks of quaternary ammonium group [-N^+^–(CH_3_)_3_], methylene group directly connected to quaternary ammonium group [-CH_2_–N^+^–(CH_3_)_3_] and characteristic stretching vibration peaks of quaternary ammonium group were observed at 967 cm^−1^ and 1452 cm^−1^ in PAD, respectively [34,35]. In addition, the wide characteristic absorption peak near 3436 cm^−1^ was attributed to the stretching vibration of -NH_2_ group in AM monomer [36]. The characteristic absorption vibration peaks of AM, DMC and CTS were overall identified in the infrared spectrum of CTS-g-PAMD, respectively, thereby verifying the successful graft polymerization of AM and DMC monomers by CTS. Furthermore, an obvious characteristic absorption peak of CTS skeleton structure after grafting P(AM-DMC) was observed, indicating that grafting copolymerization slightly impacted the internal skeleton structure of CTS molecules and the ring-loaded molecular structure of CTS was not damaged.

#### 3.2.2. H NMR Analysis

**Figure 4:**
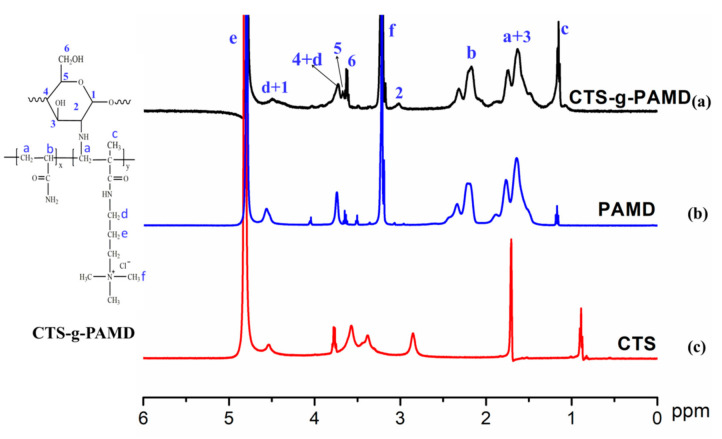
The ^1^H NMR of the polymers.

^1^H NMR is recognized as an effective method to study the chemical structure of polymer. Besides FTIR analysis of the polymer, ^1^H NMR of CTS-g-PAMD, PAD and CTS was studied, with the results shown in Figure 4. The formants at 1.68 ppm and 2.24 ppm express the proton chemical shifts at –CH_3_ (a) and –CH_2_– (b) on the PAD backbone, respectively. The H_c_ characteristic peak of methyl proton on quaternary ammonium group of DMC was identified at 3.19 ppm [37]. Moreover, at the chemical shift of 3.75 ppm, the proton H_d_ characteristic absorption peak of methylene directly connected to the quaternary ammonium group was observed [38]. The proton H_e_ characteristic absorption peak of methylene directly connected to the-O–C=O-branch chain was observed at 4.56 ppm [16,18,39]. The peaks of glucose ring protons H_1_, H_2_ and H_3_ on CTS were found at 4.56, 2.96 and 1.69 ppm. Glucose ring protons H_4_–H_6_ on CTS appeared at 3.82, 3.75 and 3.64 ppm, respectively. Besides CTS, characteristic proton peaks of AM and DMC were observed in CTS-g-PAMD. Accordingly, it was concluded that CTS-g-PAMD was polymerized from CTS, AM and DMC. In addition, as compared with CTS, CTS-g-PAMD hydrogen spectrum had a weakened H_2_ peak and then the peak shifted to the left inconspicuously. This is mainly due to the polymerization reaction between monomers (AM and DAC) and CTS and the grafted active site was -NH_2_ linked to C2 on CTS. The generated PAD replaced H of -NH_2_ to generate CTS-g-PAMD [31,40]. PAMD, a polymer chain formed by AM and DAC, interfered and destroyed with the H proton environment at C2 position on CTS and H proton exerted a strong meso-covering effect, thereby altering its peak intensity and affecting its position. However, the H proton peak connected C2 in CTS-g-PAMD remained obvious and its position was changed as well, verifying that the carbon skeleton structure of CTS was not broken.

#### 3.2.3. XRD and XPS Analysis

**Figure 5:**
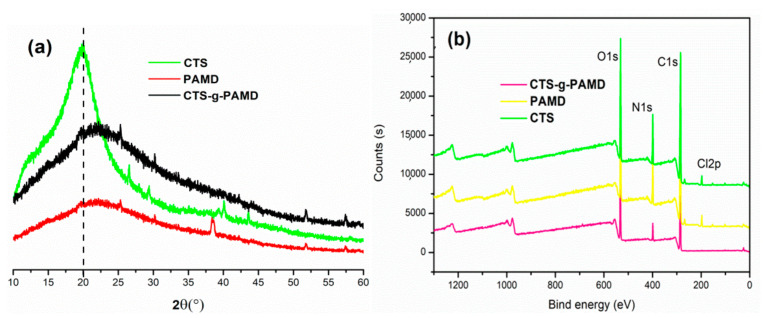
The (**a**) X-ray diffraction (XRD) and (**b**) X-ray photoelectron spectroscopy (XPS) of the polymers.

According to Figure 5a, XRD of CTS-g-PAMD, PAD, CTS was observed. At 2θ = 20°, CTS exhibited a strong dispersion peak, belonging to the typical X-ray diffraction type II mode. Compared with the XRD spectra of CTS, that of PAD displayed wider and weaker characteristic diffraction peaks with an amorphous surface morphology [41]. For CTS-g-PAMD, the characteristic diffraction peak declined and shifted to the right. The diffraction peak of CTS-g-PAMD ranged from PAD and CTS and the crystal structure of CTS-g-PAMD was similar to that of CTS. The grafting of AM and DMC onto -NH_2_, connected to C2 of CTS, led to the weakening of the ordered crystal structure of CTS, the decrease in the overall structural order of the polymer, as well as the related decrease in the crystallinity of CTS-g-PAMD. However, the ring structure of CTS was destroyed and the crystal structure was not collapsed. The grafted CTS-g-PAMD still exhibited obvious crystal structure characteristics and its crystallinity was higher than that of PAD. Figure 5b presents the full XPS spectra in CTS and CTS-g-PAMD samples. Oxygen, nitrogen, carbon and chlorine were detected in the polymerized CTS-g-PAMD sample. Chlorine originated from DMC monomer, oxygen, nitrogen and carbon come from AM and CTS. It was confirmed that cationic monomers AM and DMC were successfully followed on CTS to prepare CTS-g-PAMD.

#### 3.2.4. SEM Analysis

**Figure 6:**
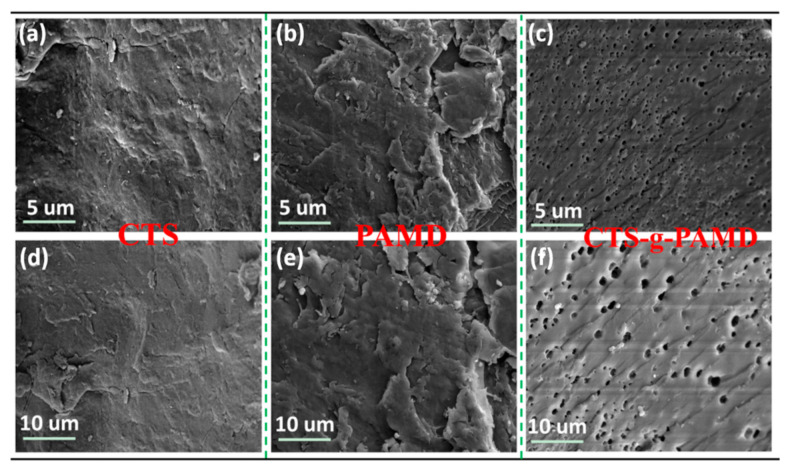
The surface morphology respectively at 8.0 kx and 4.0 kx magnification of the polymers (**a**,**d**) CTS, (**b**,**e**) PAMD and (**c**,**f**) CTS-g-PAMD.

Figure 6 significantly indicates that the surface morphology of CTS-g-PAMD, PAD and CTS also displayed obvious differences. As shown in Figure 6a, the surface of CTS was relatively smooth, with fewer holes and protrusions and hole-like structures. The layered stacking structure was relatively unapparent. In Figure 6b, the surface structure of PAD was rough and not uniform. There was a multi-layer folded structure with large surface bulge. After AM and DMC were grafted onto CTS, the surface morphology of polymer CTS-g-PAMD was obviously inconsistent with that of the other two flocculants. As shown in Figure 6c, that CTS-g-PAMD displayed a rough surface and considerable protrusions and porous structure. After AM and DMC were grafted onto CTS by radical initiation polymerization of UV-H_2_O_2_, the original structure and physical and chemical structure of CTS crystal were destroyed, resulting in a decrease in crystal structure orderliness. As a result, it increased the surface roughness of the flocculant [42]. In addition, the porous convex structure of CTS-g-PAMD provided large specific surface area and it could be easy to bind water molecules. Compared with CTS, its solubility was significantly improved and its flocculation performance was enhanced.

## 4. Flocculation

### 4.1. Effect of Flocculant Dosage

**Figure 7:**
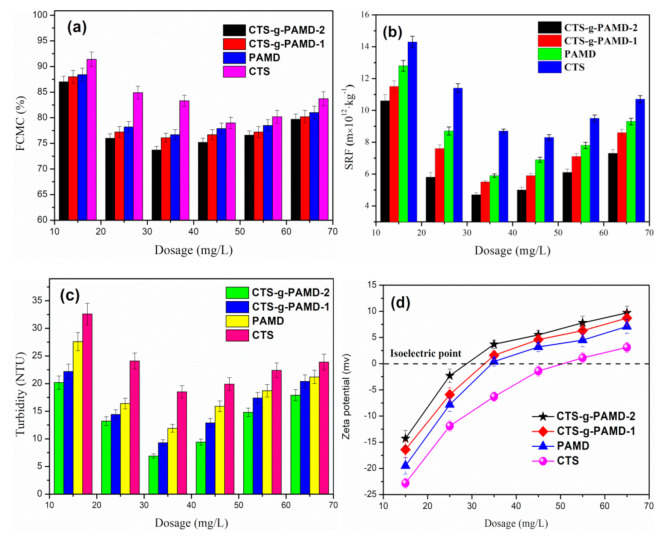
The effect of flocculant dosage on the sludge flocculation performance: (**a**) FCMC, (**b**) SRF, (**c**) turbidity and (**d**) zeta potential.

In Figure 7, the effects of four flocculants (CTS-g-PAMD-1, CTS-g-PAMD-2, PAD, CCPAM and CTS) on sludge FCMC, SRF, turbidity and zeta potential were investigated. The effect of four kinds of flocculant dosages on FCMC, SRF and turbidity displayed the same trend with the increase in flocculant dosages. With the increase in flocculant dosages, it first decreased and then increased, while zeta potential was on the rise all the time. Compared with the other three flocculants, CTS-g-PAMD-2 exhibited better flocculation performance in the whole dosage range (15–65 mg·L^−1^). Thus, it was proved that CTS-g-PAMD-2 shows a wider market application prospect. When the dosage of CTS-g-PAMD-2 was 35 mg·L^−1^, the sludge FCMC, SRF and turbidity reached the minimum (FCMC: 73.7%; SRF: 4.7 × 10^12^ m·kg^−1^, turbidity: 9.4 NTU). The surface of sludge particles was mostly negatively charged, while CTS-g-PAMD-2 molecular chain had considerable positive charges. Accordingly, with the addition of positively charged flocculant, negatively charged sludge particles were adsorbed on the flocculant as impacted by electric neutralization, electric patch and electrostatic attraction and small sludge flocs gathered to form large flocs and settle under the strong bridging and net catching effect generated by polymer chains [43].

The electric neutralization, electric patch and electrostatic attraction of flocculant were of critical importance [38]. Compared with PAD, CTS and CTS-g-PAMD-1, CTS-g-PAMD-2 contained both positive DMC and CTS monomers, exhibiting the strongest ability of electric neutralization and electric patch and better sludge dewatering effect. CTS-g-PAMD flocculant with AM and DMC monomers grafted with CTS contained longer molecular chain and more branches, thereby producing strong bridging net catching effect and making small sludge flocs aggregate to form large flocs and settle. However, CTS produced smaller flocs and longer settling performance for its small MW, low charge intensity and insufficient flocculation ability. The small flocs produced could be easy to block holes during filtration, increasing SRF, FCMC and turbidity to have a poor flocculating effect. However, too high a flocculant dosage facilitates the improvement of sludge dewatering and conditioning. With the dosage of flocculant exceeding 35 mg·L^−1^, the dewatering effect of sludge was weakened by continuously increasing the dosage and the excessive dosage caused the surface of sludge colloidal particles to be covered by superfluous polymer chains, reduced the number of exposed active adsorption sites and did not exploit the function of adsorption bridging. In addition, the charge repulsion force between sludge colloid particles wrapped with too many polymer chains led to the stabilization phenomenon of unstable colloid particles again and finally increased the difficulty of sludge dewatering [44].

### 4.2. Effect of pH

**Figure 8:**
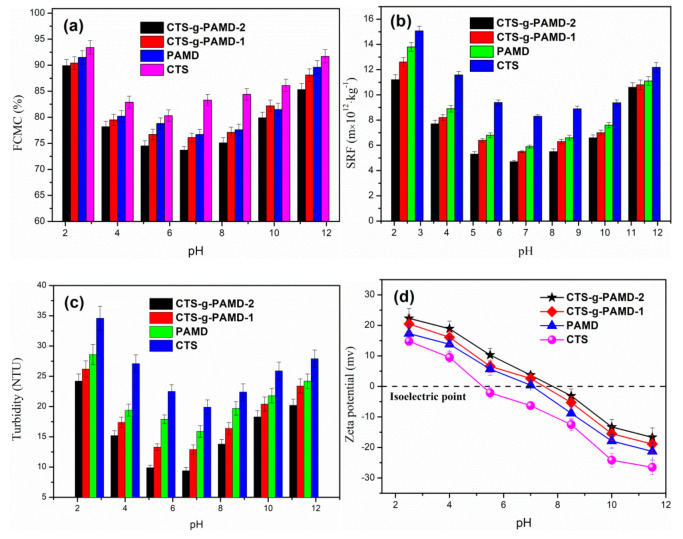
The effect of pH on the sludge flocculation performance: (**a**) FCMC, (**b**) SRF, (**c**) turbidity and (**d**) zeta potential.

The pH value of sludge solution critically impacted the sludge dewatering process. On the one hand, the surface physical and chemical properties of flocculant were significantly affected by pH [45]. On the other hand, under the presence of considerable extracellular polymers on the surface of sludge colloid particles, its chemical properties were easily affected by pH. Thus, the effect of pH on flocculating effect should be studied. Figure 8 shows the effect of pH on FCMC, SRF, turbidity and zeta potential. With pH increasing from 2.5 to 11.5, FCMC, SRF and turbidity decreased first and then increased, while zeta potential decreased constantly. 

Under different pH values, each flocculant showed different flocculating effect, proving that pH value significantly impacted flocculating effect. Under the pH of 7.0, the sludge dewatering effect was better, while strong acid and strong alkali were not conducive to fully exploit the sludge dewatering and conditioning performance of flocculant. This is primarily because strong acid and strong alkali will increase the charge strength on the surface of colloidal particles and produce strong repulsion between colloidal particles [46]. Accordingly, the colloidal particles adsorbed on the molecular chain would escape the shackles of the polymer chain and re-enter the solution and the originally destabilized sludge colloid would be stabilized again. Thus, it could hinder the flocculant to generate a large and dense floc structure and lower the turbidity through adsorption bridging. When compressing, it was easy to deform and block holes under pressure, thus increasing the sludge ratio group and reducing the sludge dewatering performance. The sludge dewatering effect of CTS-g-PAMD outperformed that of PAD and CTS. It was proved that CTS-g-PAMD shows wide applications. CTS is relatively poor in solubility, easy to contain in acidic and weakly acidic conditions, whereas it is difficult to contain in alkaline conditions. Graft copolymerization destroyed the original structure of CTS and introduced soluble AM and DMC monomers, which significantly improved its solubility and could still exert strong sludge dewatering performance under acidic or alkaline conditions [47]. CTS involved in the next copolymerization exhibited positive electrical properties, helping to improve the charge neutralization and patching ability of CTS-g-PAMD. CTS-g-PAMD with rough surface and enough porous junctions could quickly absorb water and dissolve and its molecular chain stretched well, which was easy to play a strong adsorption bridging role. CTS-g-PAMD exhibited better sludge dewatering performance in a wide pH window (pH = 4.0–10.0), demonstrating that CTS-g-PAMD shows bright market application prospects.

### 4.3. Effect of CTS Grafting Efficiency

**Figure 9:**
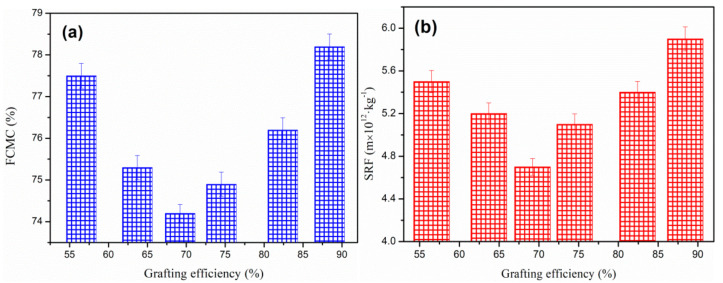
The effect of flocculant GE on the sludge flocculation performance: (**a**) FCMC and (**b**) SRF.

In Figure 9, the effect exerted by CTS grafting efficiency on sludge dewatering effect was investigated. FCMC and SRF were found to both decrease first and then increase with the change of grafting efficiency.

When the CTS grafting efficiency was 69.5%, the sludge dewatering effect of CTS-g-PAMD reached a good level. CTS-g-PAMD with appropriate CTS grafting efficiency is required to maintain an efficient sludge dewatering and conditioning effect. On the one hand, it could ensure that it contains sufficient CTS and DMC monomers, capable of significantly enhancing its charge neutralization ability and able to completely neutralize more negative sludge particles [48]. Under adsorption bridging, it helps generate large and dense sludge flocs. On the other hand, it could ensure that the amount of grafted AM and DMC monomers of high reactivity is sufficient and CTS-g-PAMD exhibiting large MW is easy to be generated, facilitating the occurrence of adsorption bridging. However, CTS grafting efficiency was too high, exerting adverse effects. Excessive CTS content would cause the relative decrease of AM and DMC content. During polymerization, the meta-steric hindrance was relatively large, the MW of synthesized CTS-g-PAMD decreased and the adsorption bridging effect was significantly reduced. In addition, too high CTS grafting efficiency would generate excessive positive charge and result in strong charge repulsion, which hinders colloid destabilization. Accordingly, CTS-g-PAMD with CTS grafting efficiency of 69.5% is more suitable and easier for sludge dewatering and conditioning.

### 4.4. Characteristics of Sludge Flocs

#### 4.4.1. Sludge Flocs Size and Fractal Dimension

**Figure 10:**
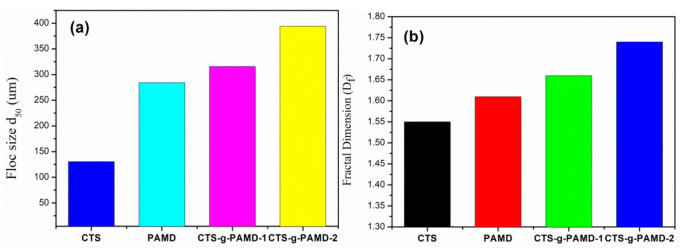
(**a**) Floc size d_50_ and (**b**) floc D_f_ for the polymers.

Since the floc structure characteristics of sludge were closely related to dewatering effect, the particle size and fractal dimension of conditioned sludge should be explored. Figure 10 shows the particle size distribution and fractal dimension of sludge flocs corresponding to CTS-g-PAMD-2, CTS-g-PAMD-1, PAD and CTS under the optimal flocculation conditions (pH = 7.0, Dosage = 35 mg·L^−1^). Among the four flocculants, CTS-g-PAMD-2 exhibited the largest flocculant size and fractal dimension (d_50_ = 379.142μm, D_f_ = 1.58), demonstrating that CTS-g-PAMD-2 exerted the best flocculation conditioning effect on sludge. CTS and DMC in CTS-g-PAMD-2 had high positive charges, capable of enhancing the ability of charge neutralization and patching, as well as of expediting the destabilization of negatively charged sludge particles and facilitating sludge dewatering. The MW of CTS-g-PAMD-2 formed by grafting AM and DMC with CTS was larger. During flocculation, its molecular chain had better linear distribution and extension; it could also adsorb more sludge particles through adsorption bridging effect and act as bridging effect between particles or between particles and flocs to generate flocs with larger particle size. Compared with the other three flocculants, CTS-g-PAMD-2 exhibited strong ability of electric neutralization, electric patch and adsorptive bridging. More negatively charged particles were neutralized and adsorbed on the molecular chain. Under the action of shear stress generated by flocculation agitation, the mentioned flocs wrapped by the molecular chain were continuously squeezed, the gaps existing in them were continuously packed and the structure turned out to be increasingly compact, finally forming a large and compact sludge flocs structure. Accordingly, the mentioned large and compact sludge flocs facilitated sludge-water separation and they could significantly reduce SRF and FCMC and significantly improve the sludge conditioning and dewatering effects [49].

#### 4.4.2. Floc Formation, Breakage and Regrowth 

**Figure 11:**
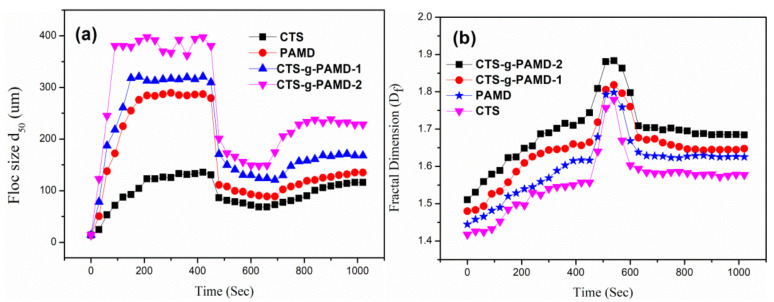
Floc formation, breakage and regrowth process at 400 r/min: evolution of (**a**) floc size and (**b**) fractal dimension over time.

Sludge flocs size and fractal dimension investigation is at the optimal flocculation conditions (pH = 7.0, Dosage = 35 mg·L^−1^). During the actual flocculation, the generated sludge flocs were easily interfered by external forces (vibration, swaying, shearing, etc.), so the breakage and regrowth of the generated flocs should be explored. In Figure 11, the floc particle size (d_50_) gradually increased to a stable state after the initial flocculation, then a sharp decrease in the floc particle size after the crushing and then a gradual recombination to a stable state during the flocculation stage after the breakage. However, the D_f_ of the four flocculants increased rapidly after crushing; then, it decreased to a stable value state in the regenerated state. 

It was found that the particle size of sludge flocs corresponding to the four flocculants decreased significantly under the larger stirring speed. This is because the shear force generated by rapid stirring completely destroys the original floc structure and the particle size of the produced floc was reduced [50,51]. After the slow stirring stage after crushing, the destroyed coal flocs grew again and reached a stable size, proving that the flocs had secondary growth phenomenon after crushing. For D_f_, under the action of large shear force, the mentioned flocs were destroyed and a smaller and denser structure was formed. In addition, in the process of stirring and crushing, the original floc structure could reconstruct a denser floc structure again, which also increased their D_f_ values [52]. However, no matter what type of flocculant was used, it would be difficult for the flocs to be completely regenerated to the size before crushing, indicating that the crushing and regeneration of flocs is an irreversible process [53,54]. It is clear that compared with the other three flocculants, the flocs corresponding to CTS-g-PAMD-2 has the largest particle size before and after crushing and the crushed coal fragments still have strong renewable property and can quickly gather together to form a large and dense flocs particle. When the shear force generated by rapid stirring will significantly destroy the bridging effect between the original flocs and the bridging and adsorption effect between the flocs by the polymer chain will rapidly weaken, at this time, the electric neutralization and adsorption effect will play a dominant role in the flocculation process [55]. As cationic CTS and DAC in CTS-g-PAMD-2 significantly enhance the charge neutralization, electrostatic and bridging adsorption of flocculant, once the flocs are destroyed by large shear force, the cationic structures accumulated on small fragments of sludge flocs will recombine under the action of charge neutralization and adsorption capture and aggregate again to generate larger dense flocs. Thus, using CTS-g-PAMD-2 for conditioning can quickly generate a large and dense floc structure with strong resistance to external force damage and regeneration capability, which significantly improves the sludge-water separation effect of sludge floc particles. Because the actual flocculation experiment is subject to many external interference factors, the flocs are easily disturbed by external forces and the generated flocs are easy to break. At this time, the new CTS-g-PAMD-2 will have obvious advantages.

#### 4.4.3. Sludge Flocculation Mechanism

**Figure 12:**
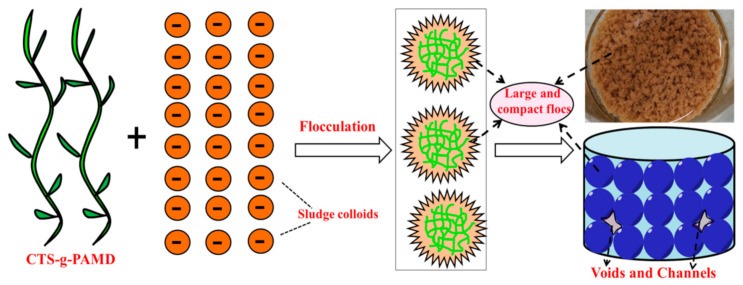
The flocculation mechanism of the CTS-g-PAMD.

Based on the analysis results of sludge flocculating effect (SRF, FCMC, turbidity), zeta potential, flocs characteristics (e.g., floc size (d_50_), D_f_ and breaking and regrowth performance), the possible flocculation mechanism is summarized. Because CTS-g-PAMD contains considerable cationic DMC monomers and positively charged CTS, the positive charge density on CTS-g-PAMD increases, thus significantly improving the charge neutralization and patching ability of CTS-g-PAMD. In addition, the grafted CTS-g-PAMD has a higher MW, its molecular chain stretches and extends well in solution and the adsorption and bridging effect is also strong. Negatively charged sludge particles are completely neutralized and aggregated under the action of adsorption bridging to form a large and compact floc structure. In addition, the mentioned floc structures can resist external force damage and have strong regeneration ability. After being broken, it can still quickly gather together to form a large and dense sludge floc structure again. The mentioned large and dense flocs can support skeletons and withstand relatively high external pressure. More drained channels and voids can be stably preserved between flocs structures. Under pressure, drained channels and voids are not easy to deform and block, as shown in Figure 12. Accordingly, water is easily discharged through drained channels and voids and finally the sludge dewatering effect is evidently improved.

## 5. Conclusions

A new CTS-based graft flocculant CTS-g-PAMD was synthesized by microwave initiated polymerization using AM, DMC and CTS as polymerization monomers. The optimal conditions were obtained at UV power of 160 W, UV irradiation time of 50 min, H_2_O_2_ concentration of 0.08 wt%, pH of 4.5, overall monomers mass fraction of 30 wt% and m_CTS_:(m_Total Monomors_) = 20%. Under the mentioned conditions, the MW and CTS grafting efficiency are the desirable. The structure and morphology of CTS-g-PAMD were analyzed by FTIR, ^1^H NMR, XRD, SEM and XPS and their results confirmed the successful synthesis of CTS-g-PAMD. Under the initiation of UV-H_2_O_2_, AM and DMC are grafted on -NH_2_ linked to C2 of CTS, which affects the ordered structure of CTS crystal structure. Compared with CTS and PAD, the dewatering conditioning performance of grafted CTS-g-PAMD is improved. Under the optimal flocculation conditions (pH = 7.0, dosage = 35 mg·L^−1^), the SRF, FCMC and turbidity of the conditioned sludge reached 4.7 × 10^12^ m·kg^−1^, 73.7% and 9.4 NTU, respectively and the sludge dewatering and flocculating effects were ideal. CTS-g-PAMD-2 showed excellent sludge dewatering performance in a wide pH range (pH = 4.0–10.0). Zeta potential analysis shows that CTS-g-PAMD-2 contains considerable cationic DMC monomers and positively charged CTS and the positive charge density increases significantly, thus noticeably improving its charge neutralization and patching ability. In addition, CTS-g-PAMD-2 has a large MW and its adsorption bridging effect is robust as well. After conditioning, sludge can form a large and compact floc structure. This is conducive to reducing SRF, FCMC and turbidity and the enhanced sludge dewatering and conditioning effect is obvious. Moreover, the floc structure conditioned by CTS-g-PAMD-2 can resist the damage of external force and has strong regeneration ability. After crushing and breakage, the mentioned flocs can still quickly gather together to form a large and dense sludge flocs structure again. More drained pores and voids can be formed between the mentioned floc structures. Under pressure, water is easily discharged through the mentioned pores and voids and finally the sludge dewatering effect is significantly improved.

## Figures and Tables

**Table 1:** The details of the active sludge used in the experiment.

pH	Density (g·mL^−1^)	Zeta Potential (mV)	SRF (×10^12^ m·kg^−1^)	Moisture (%)	VSS/TSS	Appearance
7.3	1.018	−25.48	44.7	97.9	0.714	Yellow and fresh

## Supplementary Materials

The following are available online at https://www.mdpi.com/2073-4360/12/11/2738/s1, Text S1: The measuring method for the polymer molecular weight, Text S2: Analytical methods for the fractal dimension Df of the flocs, Text S3: Analytical methods for FCMC and SRF. References [11,21,22,23] are cited in the Supplementary Materials.
