# Supplementary material for: Chitosan Modified Cationic Polyacrylamide Initiated by UV-H2O2 for Sludge Flocculation and New Insight on the Floc Characteristics Study
Source: Polymers (Basel). 2020 Nov 18;12(11):2738. doi: 10.3390/polym12112738 (PMC7698928; doi:10.3390/polym12112738)
Supplement: Supplementary file 1 [file polymers-12-02738-s001.pdf]

polymers

Supplementary Materials

Contents:

Text S1. The measuring method for the polymer molecular weight.

Text S2. Analytical methods for the fractal dimension D_f of the flocs.

Text S3. Analytical methods for FCMC and SRF.

Text S1. The measuring method for the polymer molecular weight.

The molecular weight of the polymer was calculated by the equation displayed as follows (Equation (1)).

$$M_r = 802\ [\eta]^{1.25,} \quad (1)$$

where M_r and *[η]* are the molecular weight and the intrinsic viscosity of the polymer, respectively. The intrinsic viscosities of polymers (*η*) related to the molecular weight was used to assess the absorption and bridging ability. This was conducted on an Ubbelohde viscosity meter (Shanghai Shenyi Glass Instrumental Co. Ltd., Shanghai, China) in a 2 mol L^{-1} NaCl aqueous solution at 30 °C.

Text S2. Analytical methods for the fractal dimension D_f of the flocs

Fractal dimension (D_f) could be determined by the light scattering method from the negative slope of log-log plot by a well-known power-law relation [1,2]:

$$I \propto Q^{D_f}, \quad (2)$$

where *I* is the light intensity, and *Q* is the scatter vector which can be given according to the following equation:

$$\frac{4\pi n \ \sin(\theta/2)}{\lambda}, \quad (3)$$

where *λ* is the wavelength of radiation in vacuum (631 nm in this work), *n* is the refractive index of the dispersion medium (1.33 for water), and *θ* is the scattering angle, respectively.

Text S3. Analytical methods for FCMC and SRF.

After a 30 min settling period, the conditioned sludge was poured into a Buchner funnel for filtering under a vacuum pressure of 0.09 MPa for 30 min or until the vacuum could not be maintained (in < 30 min). FCMC was obtained by the Equation (4) [3]:

$$\text{FCMC\%} = \frac{M_1 - M_2}{M_1}, \quad (4)$$

where *FCMC* is the filter cake moisture content, M_1 is the weight of the wet filter cake after filtration, and M_2 is the weight of filter cake after drying at 105 °C for 4 h. *SRF* was calculated from the Equation (5) [4]:

$$\text{SRF} = \frac{2bpA^2}{\mu c}, \quad (5)$$

where *SRF* is the specific resistance to filtration, *P* is the filtration pressure (N/m^2), *A* is the filtration area (m^2), *μ* is the viscosity of filtrate (N·s/ m^2), *b* is the slope obtained from the plot of $t/V_f(y) - V_f(x)$, where V_f is the volume of filtrate (m^3) and *t* is the filtration time (s), and filtrate volume was recorded

at 5 s, 10 s, 20 s, 30 s, 40 s, 50 s, 60 s, 70 s and 80 s during the filtration, and *c* is the weight of solids per unit filtrate volume (kg/m^3), c= [(1/C_i)/[(100C_i-C_f)/100C_f], where C_i is the initial moisture content (%) and C_f is the final moisture content (%).

References

1. Greenwood, J.; Rainey, T.; Doherty, W. Light scattering study on the size and structure of calcium phosphate/hydroxyapatite flocs formed in sugar solutions. *J. Colloid Interface Sci.* **2007**, *306*, 66–71.
2. Jarvis, P.; Jefferson, B.; Parsons, S.A. Breakage, regrowth, and fractal nature of natural organic matter flocs. *Environ. Sci. Technol.* **2005**, *39*, 2307–2314.
3. Wang, D.F.; Zhao, T.Q.; Yan, L.Q.; Mi, Z.M.; Gu, Q.; Zhang, Y.M. Synthesis, characterization and evaluation of dewatering properties of chitosan-grafting DMDAAC flocculants. *Int. J. Biol. Macromol.* **2016**, *92*, 761–768.
4. Zhang, H.; Yang, J.K.; Yu, W.B.; Luo, S.; Peng, L.; Shen, X.X.; Shi, Y.F.; Zhang, S.N.; Song, J.; Ye, N.; et al. Mechanism of red mud combined with Fenton's reagent in sewage sludge conditioning. *Water Res.* **2014**, *59*, 239–247.

Publisher's Note: MDPI stays neutral with regard to jurisdictional claims in published maps and institutional affiliations.

© 2020 by the authors. Licensee MDPI, Basel, Switzerland. This article is an open access article distributed under the terms and conditions of the Creative Commons Attribution (CC BY) license (http://creativecommons.org/licenses/by/4.0/).
